# Coarse Grained Modeling of Multiphase Flows with Surfactants

**DOI:** 10.3390/polym14030543

**Published:** 2022-01-28

**Authors:** Thao X. D. Nguyen, Tuan V. Vu, Sepideh Razavi, Dimitrios V. Papavassiliou

**Affiliations:** School of Chemical, Biological, and Material Engineering, University of Oklahoma, Norman, OK 73019, USA; Xuan.Duy.Thao.Nguyen-1@ou.edu (T.X.D.N.); tuanvu@ou.edu (T.V.V.)

**Keywords:** coarse grained computations, surfactants, oil–water interfaces, multiphase flow

## Abstract

Coarse-grained modeling methods allow simulations at larger scales than molecular dynamics, making it feasible to simulate multifluid systems. It is, however, critical to use model parameters that represent the fluid properties with fidelity under both equilibrium and dynamic conditions. In this work, dissipative particle dynamics (DPD) methods were used to simulate the flow of oil and water in a narrow slit under Poiseuille and Couette flow conditions. Large surfactant molecules were also included in the computations. A systematic methodology is presented to determine the DPD parameters necessary for ensuring that the boundary conditions were obeyed, that the oil and water viscosities were represented correctly, and that the velocity profile for the multifluid system agreed with the theoretical expectations. Surfactant molecules were introduced at the oil–water interface (sodium dodecylsulfate and octaethylene glycol monododecyl ether) to determine the effects of surface-active molecules on the two-phase flow. A critical shear rate was found for Poiseuille flow, beyond which the surfactants desorbed to form the interface forming micelles and destabilize the interface, and the surfactant-covered interface remained stable under Couette flow even at high shear rates.

## 1. Introduction

The interfacial behavior of immiscible fluids is important for understanding, predicting, and controlling systems that involve oil–water or oil–gas flows. Multilayer coating applications, separation applications, environmental remediation of contaminated regions in the subsurface, and petroleum engineering applications for oil mobilization are examples where such flow systems are important [1,2,3,4]. In addition to experiments, computations that are focused on the molecular scale such as molecular dynamics (MD) simulations can be used as tools to probe multifluid flows, flows with large molecules (i.e., polymers or surfactants), particulate flows, and interfacial phenomena [5,6,7,8]. However, MD techniques are computationally expensive and even unfeasible when investigating large scale systems or systems with very large molecules, because of the very small length and time scales employed (on the order of Angstroms and femtoseconds, respectively).

In contrast to MD, coarse-grained modeling techniques such as dissipative particle dynamics (DPD) can meet the need for larger particle sizes and longer time steps [9]. Coarse-graining can provide a bridge between modeling and real world applications [10]. In DPD, several atoms or molecules are consolidated in groups called *beads* that interact with each other based on Newton’s equation of motion. The forces acting on these beads depend on the type of molecules of the system. In non-equilibrium computations, when there is a flow, the theoretically expected behavior (such as the satisfaction of no-slip boundary conditions at the fluid–solid interfaces) and the fluid physical properties (such as viscosity) should be replicated with fidelity by the computational results. For flow over a flat plate, a computational protocol has been developed to satisfy the no-slip boundary condition [4,11,12,13], which could not be obtained with DPD, unless the equivalent force between the wall and the fluid DPD beads was adjusted. Additional issues emerge when considering dynamic behavior for flow of multiple fluids, since the fluid beads interact with beads of the same fluid and also with beads of the other fluid phase. In multifluid flow cases, accurate representation of the viscosity of each fluid is imperative, and a systematic determination of the DPD model parameters that dictate the macroscopic viscous behavior of the fluids in such systems is needed [14]. Detailed computational protocols for obtaining these DPD model parameters are not common in the DPD literature.

In this work, we focused on the velocity distribution of two immiscible fluids (heptadecane and water) in the presence of surface-active molecules (surfactants). One of the DPD model parameters, the dissipative parameter, determines the friction between interacting DPD beads, between beads of the same fluid, between beads of different fluids, and between the fluids and the solid walls. Poiseuille flow and Couette flow were used to compare fluid velocities to theoretical expectations and to verify the computational results. In addition, after the model interaction parameters were obtained, we computed flow fields in the presence of surfactants at the oil–water interface. Two types of common surfactants were added at the oil–water interface. Surfactants are widely used in enhanced oil recovery (EOR), especially when their cost is low and when they exhibit low adsorption on the surface of hydrocarbon reservoir rocks. They can also be used for the remediation of contaminated regions of soils. Nonionic surfactants exhibit salinity tolerance, and they are often used as co-surfactants with an anionic surfactant [15]. Herein, we investigated the effect of sodium dodecylsulfate (SDS, an anionic surfactant) and octaethylene glycol monododecyl ether (C12E8, a nonionic surfactant) on the oil–water interface. In particular, SDS represented the case of a short surfactant molecule on the interface, while C12E8 was a longer molecule. Both of these surfactants have been employed in our prior work for oil–water–nanoparticle computations [16,17], and have been employed in industrial oil–water systems such as flooding of oil reservoirs for EOR [18,19].

The contributions of this manuscript are in (a) developing a detailed procedure for the modeling of flow of two multiphase flows with a coarse-grained approach; (b) demonstrating the effects of DPD model parameters on the physical properties of multifluid systems; and (c) probing the effects of surfactants at the oil–water interface and the interfacial instability under shear that leads to micelle formation in the bulk of one of the two phases.

## 2. Materials and Methods

### 2.1. Dissipative Particle Dynamics

As mentioned earlier, every particle (or bead) in a DPD model represents a small cluster of molecules, instead of a single atom or a single molecule [20]. The force that two DPD beads *i* and *j* exert on each other consists of three components: the conservative ($F_{ij}^{C}$), dissipative ($F_{ij}^{D}$), and random ($F_{ij}^{R}$) forces [21]. These forces are pairwise additive and have the following form

(1) $$F_{ij}^{C}=\left\{\begin{array}{c} a_{ij}\left(1-\frac{r_{ij}}{r_{c}}\right){\;\hat{r}}_{ij}\;\mathrm{for}\;r_{ij}<r_{c}\\ 0\;\;\;\;\;\;\;\;\;\;\;\;\;\;\;\;\;\;\;\;\;\;\;\;\;\;\;\;\mathrm{for}\;r_{ij}\geq r_{c} \end{array}\right.$$

(2) $$F_{ij}^{D}=-\gamma w^{D}\left(r_{ij}\right)\left({\;\hat{r}}_{ij}\cdot v_{ij}\right){\;\hat{r}}_{ij}$$

(3) $$F_{ij}^{R}=\sigma w^{R}\left(r_{ij}\right)\theta_{ij}{\;\hat{r}}_{ij}$$

The position of particle (bead) *i* is $r_{i}$ and the velocity of that particle is $v_{i}$. The relative velocity and position between two particles *i* and *j* are $r_{ij}$ and $v_{ij}$, respectively, so that in the equations above $r_{ij}=\left|r_{i}-r_{j}\right|$, ${\;\hat{r}}_{ij}\;$= ${(r}_{i}-r_{j})/r_{ij}$, $v_{ij}=v_{i}-v_{j}$, $a_{ij}$ is the maximum repulsion between a pair of interacting particles, and $r_{c}$ is the cut-off radius. The friction coefficient γ (typically called the dissipative parameter) and the noise amplitude σ determine the strength of the dissipative and random forces, respectively, and they are responsible for representing the viscous behavior of the fluid system [11]. The term $\theta_{ij}$ is a Gaussian white noise function that has the following stochastic properties

(4) $$\langle \theta_{ij}\left(t\right)\rangle =0,\;\langle \theta_{ij}\left(t\right)\theta_{kl}\left(t^{,}\right)\rangle =\left(\delta_{ik}\delta_{jl}+\delta_{il}\delta_{jk}\right)\delta \left(t-t^{,}\right)$$

where $\delta_{ij}$ is the Kronecker delta, the brackets indicate average, and t is time. Furthermore, $w^{D}$ and $w^{R}$ are *r*-dependent weight functions vanishing for $r_{ij}>r_{c\;}$. Espanol and Warren [22] showed that the weight functions and the dissipation parameters are related as follows:

(5) $$w^{D}\left(r\right)={\left[w^{R}\left(r\right)\right]}^{2}$$

and

(6) $$\sigma^{2}=2\gamma k_{B}T$$

where T is the system temperature, and $k_{B}$ is the Boltzmann constant. Finally, the weight function is determined as

(7) $$w^{D}\left(r_{ij}\right)=\left\{\begin{array}{ccc} {\left(1-r_{ij}\right)}^{2}\;, & & \mathrm{for}\;r_{ij}<r_{c}\\ 0, & & \mathrm{for}\;r_{ij}\geq r_{c} \end{array}\right.$$

The time evolution of the velocity and position of each DPD particle can be calculated by Newton’s equation of motion

(8) $$\frac{dr_{i}}{dt}=v_{i}\;,$$

(9) $$m_{i}\frac{dv_{i}}{dt}=f_{i}=\sum_{j\neq i}(F_{ij}^{C}+F_{ij}^{D}+F_{ij}^{R})$$

where $r_{i}$, $v_{i}$ and $m_{i}$ are respectively the position and velocity vectors, and the mass of bead *i,* while $f_{i}$ is the inter-particle force vector acting on the particle *i.*

### 2.2. Velocity Distribution of Two Immiscible Liquids in Nanoslits

**Figure 1:**
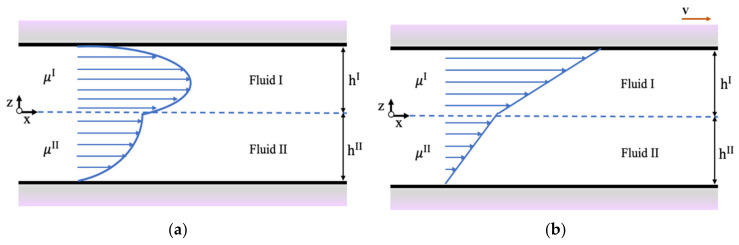
Flow of two immiscible fluids between a pair of plates under (**a**) Poiseuille, and (**b**) Couette flow conditions.

While a coarse graining model is a molecular scale model, its results should agree with theoretical and experimental predictions at larger scales, where the continuum assumption can be made. The system chosen to model two-phase flow is shown in Figure 1. Two immiscible fluids (fluids I and II) with different viscosities $\mu^{I}$ and $\mu^{II}$, respectively, flow in a nanoslit. One can assume that $\mu^{I}<\mu^{II}\;$and that the flow is in the x-direction between a pair of horizontal plates. The slit is filled with fluid I and fluid II with $h^{I}$ and $h^{II}$ being the corresponding heights. These can be adjusted by changing the ratio of the flow rate of fluid I to fluid II. The slit dimension in the z-direction is 2h, where h is the distance from the center of the channel to the wall. For the Poiseuille flow conditions, the driving force for the flow is a pressure gradient in the x-direction, while for the Couette flow conditions, the driving force is the shear applied to the fluids when the top wall of the nanoslit moves in the x-direction with velocity v (see Figure 1b).

The theoretical solution for this system in Poiseuille flow conditions (i.e., with Newtonian fluids, fully developed flow, steady state, $h^{I}=h^{II}$, and under the driving force of a pressure gradient) is known [23]. In the case of $h^{I}\neq h^{II}$, it is a trivial mathematical exercise to demonstrate that the fluid velocity profile is given as

(10) $$v^{I}=-\frac{\rho g}{2\mu^{I}}z^{2}-\frac{C_{1}}{\mu^{I}}z+C_{2}$$

(11) $$v^{II}=-\frac{\rho g}{2\mu^{II}}z^{2}-\frac{C_{1}}{\mu^{II}}z+C_{2}$$

where ρ is the fluid density; g is the pressure drop, which is applied in the DPD simulation as a body force in the flow direction $g_{x}$; and the constants of integration $C_{1}$ and $C_{2}$ found with the application of the no-slip boundary condition at the walls and the application of the continuity of stresses at the fluid–fluid interface are as follows:

$$C_{1}=\frac{\rho g}{2}\;\frac{{\left(h^{II}\right)}^{2}\mu^{I}-{\left(h^{I}\right)}^{2}\mu^{II}}{\mu^{II}h^{I}-\mu^{I}h^{II}}\;\mathrm{and}\;C_{2}=\frac{\rho g}{2}\;\frac{h^{I}}{\mu^{I}}\left(h^{I}+\frac{{\left(h^{II}\right)}^{2}\mu^{I}-{\left(h^{I}\right)}^{2}\mu^{II}}{\mu^{II}h^{I}-\mu^{I}h^{II}}\right)$$

In plane Couette flow, the upper wall moves at a constant velocity v, whereas the lower wall is stationary. The fluid velocity profile for Couette flow in a channel is expressed by

(12) $$v^{I}=v\left(\frac{C_{1}}{\mu^{I}}z+C_{2}\right)$$

(13) $$v^{II}=v\left(\frac{C_{1}}{\mu^{II}}z+C_{2}\right)$$

where

$$C_{1}=\frac{\;\mu^{I}\mu^{II}}{\mu^{II}h^{I}+\mu^{I}h^{II}}\;\mathrm{and}\;C_{2}=\frac{\;\mu^{I}h^{II}}{\mu^{II}h^{I}+\mu^{I}h^{II}}$$

### 2.3. Details of the Computational Model

**Figure 2:**
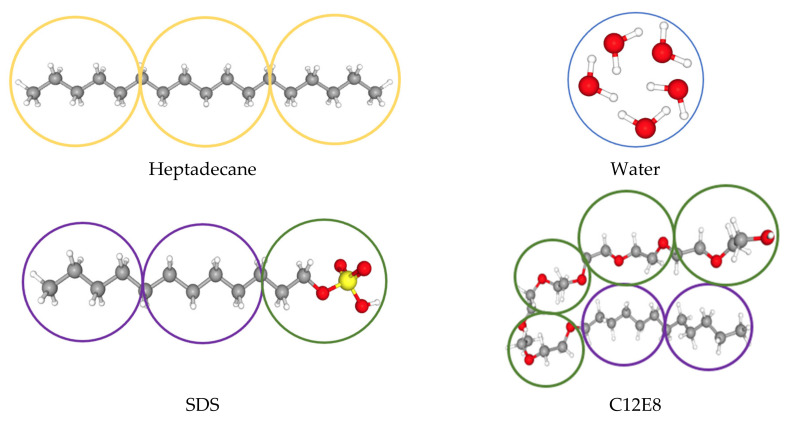
Schematic configuration of heptadecane, water, SDS, and C12E8 surfactant molecules as beads of the DPD simulations. Hydrogen, carbon, oxygen, and sulfur molecules are shown as white, gray, red, and yellow spheres, respectively. The circles represent the DPD beads that group atoms or molecules together.

All simulations were carried out using the open-source Large-scale Atomic/Molecular Massively Parallel Simulator (LAMMPS) software package [24]. In this work, five water molecules were lumped together into one water bead (W) when simulating oil–water systems and oil–water with SDS systems. The oil phase was represented by heptadecane (C_17_H_36_) molecules and each molecule included three oil beads (O) to represent the same volume of fluid as each of the water beads. The first surfactant was SDS, where each molecule contained one head (H) and two tail (T) beads, while four head and two tail beads were used for modeling C12E8 as the second surfactant. The schematic configuration of each molecule in the DPD simulations is shown in Figure 2. For the simulations of oil–water with C12E8, six water molecules were lumped in one DPD bead with the purpose of keeping comparable volumes of beads in the computations for all molecules. In addition, for the surfactant C12E8, the angle potentials that were applied between beads needed to be considered. Based on the C12E8 molecular structure, the tail–tail–head angle was fixed as 180° and all other angles were kept at 130° in order to meet the need for interfacial stability when the surfactant was added [16].

The particle mass, the temperature, and the cut-off radius ($r_{c}$) were chosen as the units of mass, energy, and length within the DPD simulations, so that *m* = *r_c_* = *k_B_ T =* 1. Since non-equilibrium computations were conducted, the temperature in the simulations was kept constant by employing the particle velocity rescaling thermostat available in LAMMPS. Every 200 time steps, the velocity of the beads was rescaled so that the temperature was kept constant at *T* = 1 (in DPD units of temperature). Furthermore, the bead number density was selected as *ρ* = 5 beads per unit volume. Using these parameters, the time scale was determined as $\tau =r_{c}\sqrt{m/k_{B}T}$ **[25]**. Conversion of time and length scales from DPD units to physical units in the case of C12E8 simulations was conducted using the same approach as for the SDS, and are shown in Table 1 [16,26].

The details for the selection of the interaction parameters $a_{ij}$ representing the repulsion between the different types of beads (water, oil, surfactant tail, surfactant head) have been published previously and are adopted herein [16]. Groot and Warren [21] have shown that the repulsion parameters between the beads of the same fluid (same type of beads should be

(14) $$a_{ii}=75\frac{k_{B}T}{\rho }$$

Therefore, when the number density is ρ=5 and $k_{B}T=1$, the value of the interaction parameter is $a_{ii}=15$. All the interaction parameters between oil, water, and wall beads are listed in Table 2 for simulations with oil and water only [16].

While the repulsion interactions between beads (i.e., the repulsion parameter in Equation (1)) depend on the type of molecules in the simulated system, the focus here was on investigating the dynamic behavior of the fluids. Regarding the DPD beads, there does not appear to be a single rule that can be used to determine their size. Instead, the physical system and the goals of the research project should help to define the size of the beads and how a molecule should be split into different beads. In the present case, where surfactant molecules are simulated in the system, the use of separate beads that represent the head and the tail behavior is needed. The main rule is that the beads for all simulated components should represent atoms or molecules that have the same volume in physical dimensions. In prior work, we have explored the effects of coarse-graining level (i.e., how many water molecules should be in a bead) on the DPD parameters of a system with water and carbon nanotubes [27]. We found that macroscopically observed hydrodynamic behavior (i.e., slip length and drag coefficient) agreed with findings from the molecular dynamics when the interaction parameters appearing in the conservative force (see Equation (1) were modified. The procedure was a trial-and-error procedure, so a general rule was not obtained. However, a larger level of coarse-graining led to larger length and time scales (for example, in [27], the maximum number of water molecules in the largest bead was 273, corresponding to beads with a diameter equal to the diameter of the carbon nanotubes at 2.5 nm). In general, increasing the size of the entities that the beads represent could affect the results, and modifications of the model parameters might be needed.

The experimentally determined micelle aggregation number for SDS and C12E8 in an aqueous solution was used to obtain the repulsion parameters for the surfactant interactions between themselves and water, and the interfacial tension (IFT) was used to obtain the oil–water interactions. The surfactant–oil interactions were obtained by matching the IFT in the DPD system at different surfactant concentrations at the interface with the experimentally obtained values of IFT as a function of the surfactant interfacial concentration. The key to that approach was to use the theoretically expected amount of surfactant molecules on the interface at the critical micelle concentration (CMC) and at fractions of the CMC, in order to obtain the IFT within a reasonable computational time. If the surface concentration was chosen randomly, the high concentration of surfactant could push some surfactant molecules out of the interface [27]. In DPD units, 1.61 SDS surfactant molecules per $1\;r_{c}^{2}$ area was used as the number for SDS molecules on the water–heptadecane interface. Similarly, 1.47 molecules per $1\;r_{c}^{2}$ area in DPD units was applied for the system of C12E8 on a water–heptadecane interface [16]. According to this procedure, the repulsion parameters used in this paper in the case of SDS at the oil–water interface are listed in Table 3 and the repulsion parameters for C12E8 are listed in Table 4.

Both Poiseuille and Couette flow simulations were performed in a rectangular box of size $20\times 20\times 21\;r_{c}^{3}$, as seen in Figure 1. The initial positions of the DPD beads were randomly selected between two parallel solid plates, and the computational domains were assumed to be periodic in the streamwise x-direction and in the spanwise y-direction. The computational domain consisted of 1250 wall beads and 41,600 fluid particles to maintain the density as 5. To determine the viscosity of two immiscible fluids under Poiseuille flow condition, a body force was chosen as $g_{x}=0.01$, while the velocity of the walls was set equal to zero. This body force is the way to introduce the pressure drop that drives the Poiseuille flow. The effect of surfactants on the velocity profile and the critical shear rate that could create micelles were also examined. Based on the calculations above, the number of surfactant molecules at the oil–water interface at the CMC were approximately 644 and 588 for SDS and C12E8, respectively, equal to 1932 and 3528 surfactant beads, respectively. The duration of the simulations was $10^{6}$ timesteps with Δt=0.02 in DPD units. Simulations were performed with $2\times 10^{6},\;3\times 10^{6}$, and$\;5\times 10^{6}$ timesteps and the results did not change—the desorbed micelles were observed to be stable in all computations. Simulations were further performed for a larger box size, with dimensions $L_{x}\times L_{y}\times L_{z}=20\times 20\times 51\;r_{c}^{3}$ to examine the influence of box size on the velocity profile and the magnitude of critical shear rate. Snapshots of the system were visualized by the visual molecular dynamics (VMD) software [28].

## 3. Results

### 3.1. Single Phase Flow, No-Slip Boundary Conditions, and Determination of Fluid Viscosity

For fluid flow in a nanoslit, the no-slip boundary condition at the walls needs to be obeyed. The solid walls were created by freezing the DPD wall beads, while bounce-back boundary conditions were used on the surface of the walls to prevent any fluid (water or oil) beads from penetrating into the solid domain. Since the soft potential used in DPD does not prevent penetration, one needs to be aware of this issue. The bounce-back condition was set at a distance of 0.1 DPD units from the inner face of each wall, so that the nanoslit height was 20.8 DPD units.

**Figure 3:**
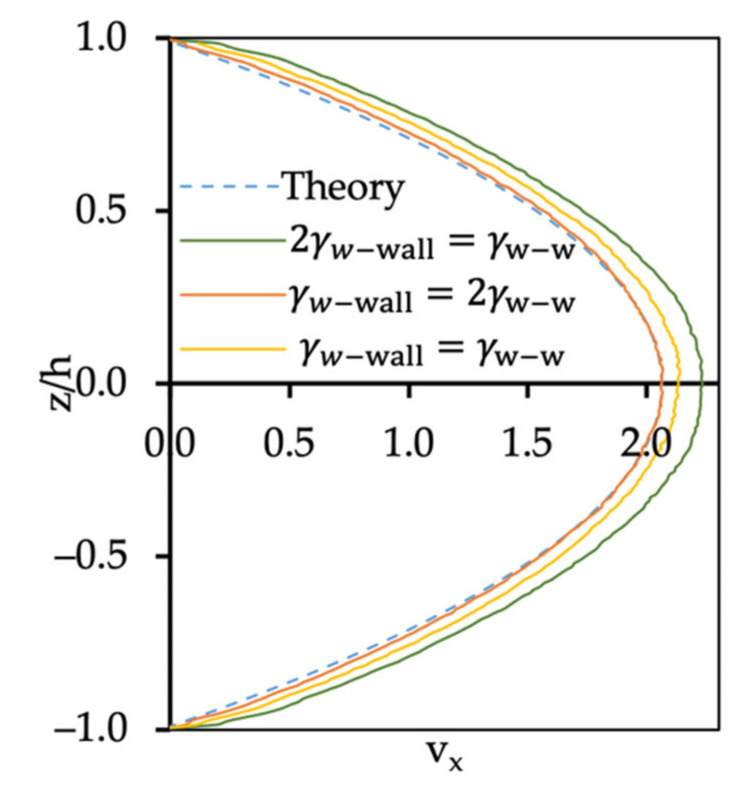
Poiseuille flow velocity profile for water with different dissipative parameter values between the wall and the water beads.

The results for the single fluid flow simulation for water are shown in Figure 3, and are compared to the Poiseuille flow equation for the fluid velocity profile (the dotted lines). First, we simulated the case with the bounce-back boundary condition and kept the dissipative parameter between water–water and between water–wall beads the same, γ=4.5. The velocity profile for water flow (yellow line) was higher than expected when compared to the parabolic Poiseuille flow solution, resulting in a large slip of the fluid at the walls. From this finding, it can be concluded that using the same friction coefficients between different types of materials would not result in an accurate representation of the system, if one wanted to prevent fluid particles from entering the wall domain. If the dissipative parameter for water–wall interactions was reduced by one-half that of water–water, the velocity profile for water flow (green line) was also greater than expected and a large slip was still present.

When the dissipative parameter for water–wall interactions was increased to twice that of water–water (red line), the no-slip condition was satisfied. The normalized error (${\bar{e}}_{L_{2}}$) can be used to quantify the difference between the theoretical and the simulation results, calculated as follows:

(15) $${\;\bar{e}}_{L_{2}}=\frac{{∥v^{\mathrm{theory}}-{\;v}^{\mathrm{simulation}}∥}_{L_{2}}}{{∥v^{\mathrm{theory}}∥}_{L_{2}}}=\frac{{\left[\frac{1}{N}\sum {\left(v^{\mathrm{theory}}-{\;v}^{\mathrm{simulation}}\right)}^{2}\right]}^{\frac{1}{2}}}{{\left[\frac{1}{N}\sum {\left(v^{\mathrm{theory}}\right)}^{2}\right]}^{\frac{1}{2}}}$$

where N is the number of observations. From Figure 3, the maximum velocity was 2.063, and the parabolic velocity profile was

(16) $$V_{x}=\frac{\rho g}{2\mu }h^{2}\left[1-{\left(\frac{z}{h}\right)}^{2}\right]=2.063\left[1-{\left(\frac{z}{10.4}\right)}^{2}\right]$$

The viscosity of water can be obtained based on the velocity maximum, so that $\mu_{water}=1.31$ in DPD units.

**Figure 4:**
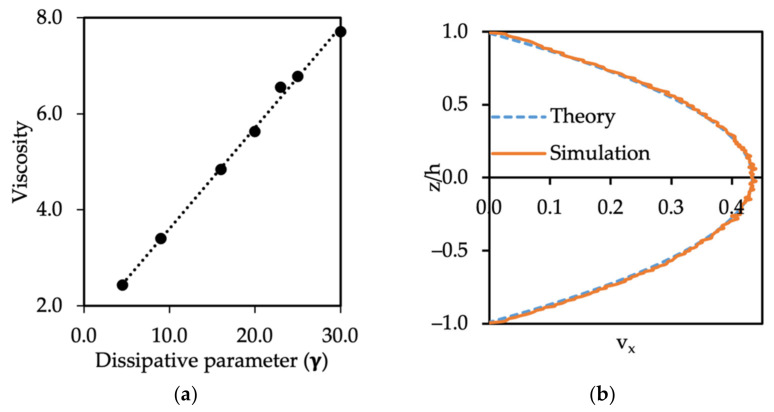
(**a**) Relation between the dissipative parameter and computed fluid viscosity, and (**b**) Poiseuille flow velocity profile for oil flow when $\gamma_{oil-oil}=22.5$ and $\gamma_{oil-wall}=45.0$.

In fact, the dynamic viscosity of water and heptadecane is $8.9\times 10^{-1}$ mPa·s and 4.21 mPa·s [29], which is equivalent to 1.31 and 6.24 in DPD units, respectively. In order to estimate the correct value of the dissipation parameter for oil flow dynamic simulations, single phase flow computations for oil in Poiseuille flow were conducted for different values of the body force $g_{x}$ acting on the oil beads. The maximum velocity of the parabolic profile was found, and from that, the viscosity of the oil phase. Finally, in Figure 4a the effect of the dissipative parameter on the oil viscosity is presented. The results of the simulations were in a good agreement with the theoretical velocity [30]. A straight line can be fitted to the simulation results, so that

(17) $$\mu_{\mathrm{oil}}=0.21\;\gamma_{\mathrm{oil}-\mathrm{oil}}+1.51$$

Therefore, the dissipative parameter for oil–oil, which yields the reported dynamic viscosity of the oil ($\mu_{oil}=6.24$) is equal to $\gamma_{\mathrm{oil}-\mathrm{oil}}=22.5$ and the dissipative parameter for the oil–wall interaction is twice that, and equal to $\gamma_{\mathrm{oil}-\mathrm{wall}}=45.0$, since we applied the same rule as for the water–wall case. The Poiseuille flow velocity profile for oil was also verified with the parabolic flow theoretical solution, as seen in Figure 4b. There was agreement with the theoretical velocity profile, and we can conclude that the dissipative parameters for a single phase of oil ($\gamma_{\mathrm{oil}-\mathrm{oil}}$) and water ($\gamma_{\mathrm{water}-\mathrm{water}}$) in the system of two immiscible fluids should be dissimilar. This is important when dynamic simulations are conducted instead of computations at equilibrium.

It is important to comment here on the Newtonian character of the results for the computations. One would expect a parabolic velocity profile for Poiseuille flow when the fluid is Newtonian. Long n-alkanes, however, exhibit non-Newtonian behavior at high shear rates. The transition to shear-thinning can be estimated based on the inverse of the alkane chain relaxation time, given as $\gamma_{c}=\frac{1}{\tau }$, where τ is the characteristic relaxation time from the Rouse model for polymer dynamics [31]. Specifically, $\tau =\frac{12M\mu }{\pi^{2}\rho RT}$ [32], where M is the molecular mass and R is the ideal gas constant. Calculations for pure heptadecane show that the relaxation time based on the Rouse model is 640 ps, meaning that the shear rate for the case of Poiseuille flow was below the critical shear rate. For Couette flow, when the velocity of the top wall of the narrow slit was 5, the shear rate was one order of magnitude above the critical shear rate. However, even in that case, a Newtonian fluid behavior was observed. The reason might be that the Rouse model and the transition to shear thinning account for the entanglement and disentanglement of the n-alkane chains. In the present case of coarse-graining, the chain of the alkane is represented with three beads, so that disentanglement of the chains does not occur.

### 3.2. Flow of Two Immiscible Fluids

**Figure 5:**
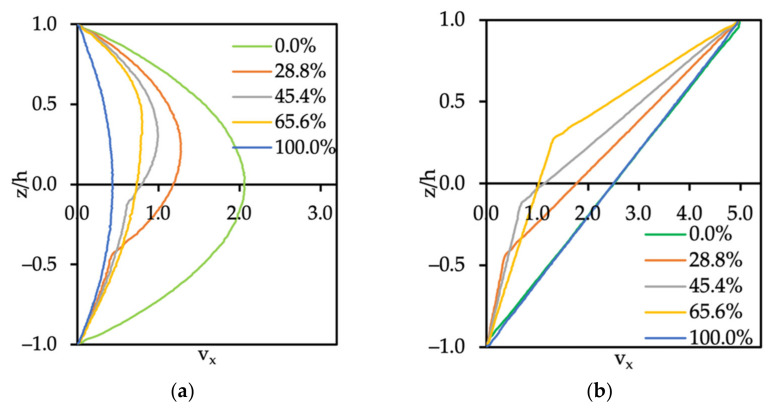
Velocity profile for two immiscible fluids with various percentages of oil (0.0%, 28.8%, 48.4%, 65.6%, and 100.0%) under (**a**) Poiseuille and (**b**) Couette flow conditions. The oil flows through the bottom side of the channel and the water through the top side of the channel.

In Figure 5, we show the velocity profile across the nanoslit when immiscible oil and water flow through it. The ratio of oil to water flow was varied from 100% of water to 100% of oil under Poiseuille and Couette flow conditions. The velocity profile shown in Figure 5a was obtained after the Poiseuille flow was fully developed. The result in the case of 45.4% of oil was in good agreement with the analytical solution of Bird et al. [23] (see Equations (10) and (11)). For the cases of 0.0%, 28.8%, 65.6%, and 100.0% of oil in the system, the slope and the shape of the velocity profiles were different at the dividing streamline between the oil and water interface. Moreover, the normalized error ${\bar{e}}_{L_{2}}$ between the theoretical and the computational results is shown in Table 5.

The Couette flow field was obtained by applying a constant force to the top wall of the nanoslit, so that the velocity of the top wall was equal to 5.0 (in DPD units), while the bottom wall was kept stationary. The same simulation parameters as for the single phase of oil and water in Poiseuille flow were applied to satisfy the no-slip boundary conditions. As expected, when the ratio of oil over water increased, the interface velocities increased (see Figure 5b). The error between the theory and computational values for both cases of Poiseuille and Couette flow is displayed in Table 5.

### 3.3. Dynamic Oil–Water System with Surfactants

**Figure 6:**
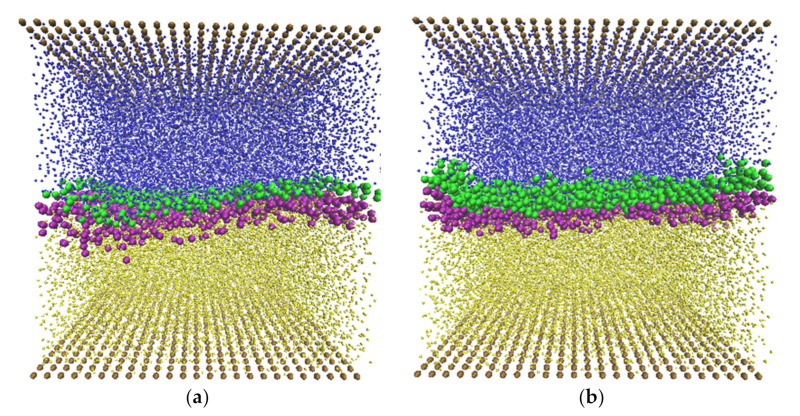
Snapshots of (**a**) SDS and (**b**) C12E8 surfactant at the oil–water interface with 50% of oil and water between two solid walls under Poiseuille flow. The wall, water, and oil beads are shown as ochre, blue, and yellow, respectively. The surfactant tails are purple, while green beads represent the head beads of the surfactants.

The two surfactants, SDS and C12E8, were selected because of their different molecular size and character, and because they have been commonly used in experiments and applications. In this work, they were used to probe the interfacial behavior of the oil–water system under shear. Figure 6 is a visual illustration of the SDS and C12E8 surfactant molecules at the oil–water interface between two parallel solid plates. The external force $g_{x}=0.01$ was applied to create the Poiseuille flow while the walls were stationary. As can be seen, all the surfactant molecules remained at the interface with the hydrophilic head beads (green beads) facing toward the aqueous phase, whereas the hydrophobic tail beads (purple beads) tended to face the oil phase.

#### 3.3.1. Velocity Profile of Oil–Water System with Surfactants

**Figure 7:**
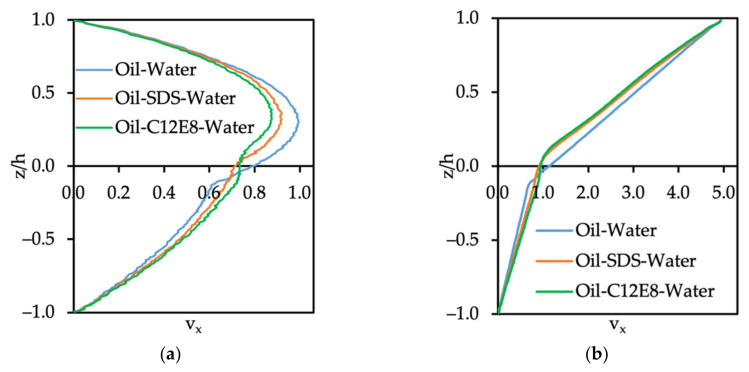
Velocity profile of oil–SDS–water and oil–C12E8-water in (**a**) Poiseuille and (**b**) Couette flows. The oil flows in the bottom side of the channel (z/h < 0) and the water flows through the top side (z/h > 0). The results are for a computational box with a size of 20$\times 20\times 21\;r_{c}^{3}$.

The influence of surfactants located at the oil–wall interface on the velocity profile under Poiseuille and Couette flow configurations is shown in Figure 7a,b, respectively. It is clearly seen that the presence of the surfactant at the interface leads to a reduction in the water phase velocity and an increased velocity of the oil phase. The most drastic change of velocity for either the Poiseuille or Couette flow conditions was observed when C12E8 was present. To clarify the contribution of the surfactant on the velocity profile, we changed the number of surfactants (both SDS and C12E8) for Poiseuille flow. In such a system, the presence of the surfactants may affect the viscosity of the oil phase, since the surfactant molecules are large. The surfactants also change the oil–water interfacial tension, but the observation that C12E8, which is the larger molecule, has the stronger effect on the velocity profile led to the hypothesis that this behavior is a viscosity-related effect. For a mixture of fluids, one can define an effective viscosity $\left(\mu_{eff}\right)$ that depends on the fluid components and their mole fraction in the mixture. The formula for $\mu_{eff}\;$of liquids incorporates the mole fraction of the fluids in the mixture and a weighted average of the viscosities [33], as follows:

(18) $$\ln\mu_{eff}=\overset{N}{\underset{i=1}{\sum }}x_{i}\ln\mu_{i}\to \ln\left(\frac{\mu_{eff}}{\mu_{2}}\right)=ax_{1}\;\ln\left(\frac{\mu_{1}}{\mu_{2}}\right)$$

If this is the case here, we would expect a linear function of the logarithm of the effective viscosity and the surfactant mole fraction in the oil phase and water phase. The effective viscosity in the oil phase and water phase was determined from the velocity profile and Equations (10) and (11). The mole fraction $x_{i}$ is the ratio between the moles of surfactant tails and oil, or the ratio between the moles of surfactant heads and water.

**Figure 8:**
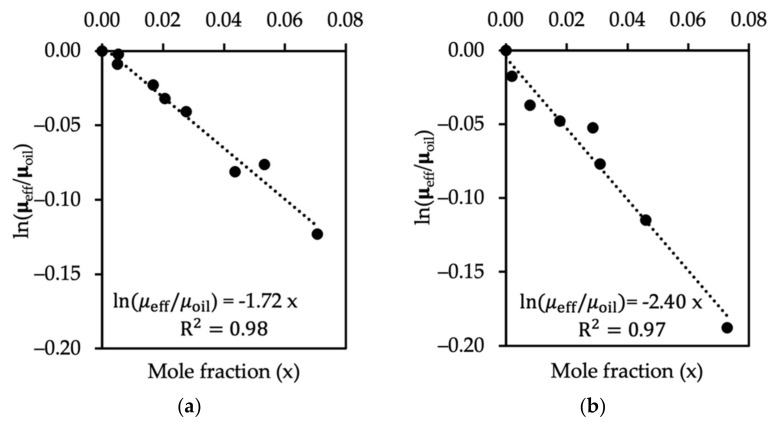

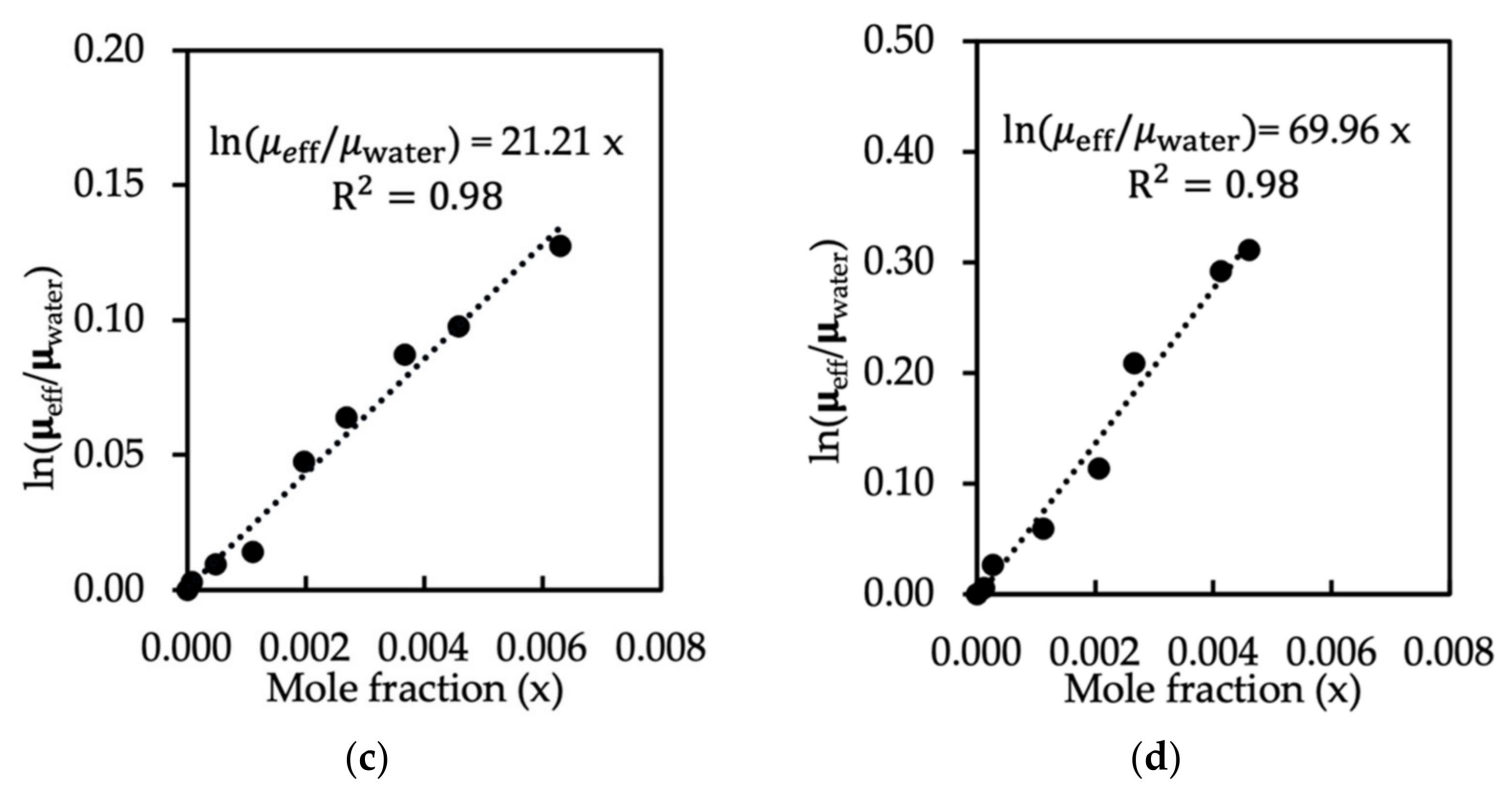
$\ln\left(\mu_{eff}/\mu_{oil}\right)$ as a function of mole fraction $x_{i}$ of (**a**) SDS and (**b**) C12E8 used to stabilize the oil–water interface in the case of the oil phase and $\ln\left(\mu_{eff}/\mu_{water}\right)$ as a function of mole fraction $x_{i}$ of (**c**) SDS and (**d**) C12E8 in the case of the water phase.

As can be seen from Figure 8a with a very high number of SDS, the effective viscosity of the oil phase decreased significantly. In this figure, results from both the small ($20\times 20\times 21\;r_{c}^{3}$) and the large ($20\times 20\times 51\;r_{c}^{3}$) computational box sizes are included. The most interesting point is that the relationship between the surfactant mole fraction and the natural logarithm of the ratio o effective viscosity to the viscosity of oil can be represented by a linear function. When the surfactant mole fraction is zero, the linear fit has to pass through the point $\mu_{eff}/\mu_{oil}=1.0$ and $\ln(\mu_{eff}/\mu_{oil})=0.0$. As the concentration of SDS increased, the effective viscosity of the oil phase decreased significantly. The investigation of effective viscosity in the case of C12E8 led to a similar conclusion (shown in Figure 8b). In addition, a linear function of $\ln(\mu_{eff}/\mu_{water})$ and mole fraction of surfactant heads in the water phase was observed in Figure 8c,d. In summary, the presence of surfactants exerts an influence on the behavior of two immiscible fluids flowing between two parallel plates. The natural logarithm between effective viscosity and the viscosity of oil or water changes linearly with the surfactant tails or heads’ mole fraction.

#### 3.3.2. Micelle Formation

**Figure 9:**
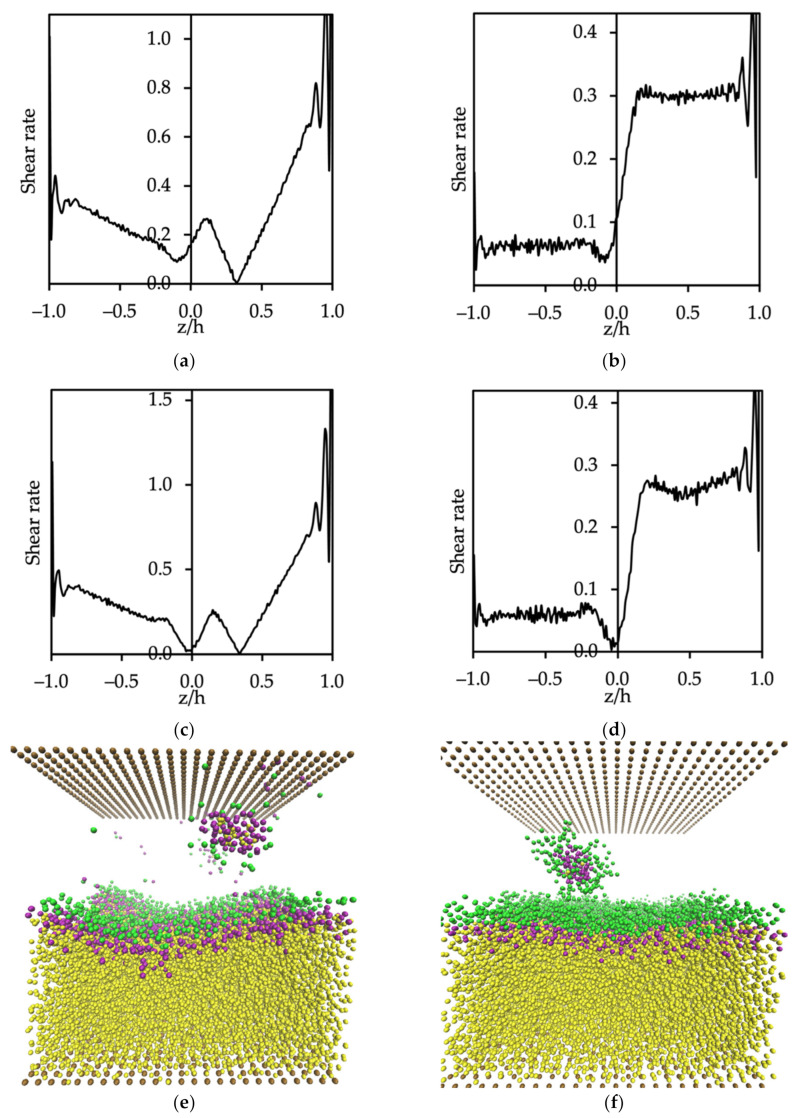
Shear rate and fluid behavior of Poiseuille flow for (**a**,**e**) oil–SDS–water and (**c**,**f**) oil–C12E8–water, and Couette flow for (**b**) oil–SDS–water and (**d**) oil–C12E8–water (box size 20$\times 20\times 21\;r_{c}^{3}$). Oil flows through the bottom of the channel and water through the top side of the channel (**a**–**d**). The color code for the DPD beads in (**e**,**f**) follows the colors in Figure 6.

Changes in the shear rate applied in the flow fields were obtained by changing the streamwise body force in Poiseuille flow and by changing the velocity of the moving wall in Couette flow. The motivation was to investigate the stability of the oil–water interface, and the results are presented in Figure 9. The shear rate, $\gamma_{S}$, is given as

(19) $$\gamma_{S}=\left|\frac{dv_{x}}{dz}\right|$$

For Poiseuille flow, the shear rate was the maximum at the top and bottom walls, and had different values in the case of multifluid flow because of the difference in the viscosity of the two fluids. The minimum shear rate was reached at the position corresponding to the maximum velocity of the water flow, which can be seen in Figure 9a,c. In addition, Figure 9e,f shows the effect of shear rate on creating micelles, when the surfactants desorbed from the oil–water interface and moved into the water phase. In the case of SDS at the CMC, there appeared to be a critical value of shear rate for the desorption of micelles, which formed when the value of the body force of Poiseuille flow $g_{x}$ was greater than 0.038. Figure 9a shows that the maximum value of shear rate at the interface was 0.265 (in DPD units), while the critical value of shear rate for the oil phase in the case of Couette flow was 0.063 (Figure 9b). In the case of C12E8, the maximum value of shear rate at the interface was 0.261 (the body force of Poiseuille flow $g_{x}=0.038\;$was shown in Figure 9c) and the critical value of shear rate for the oil phase under Couette flow was 0.059 (Figure 9d).

**Figure 10:**
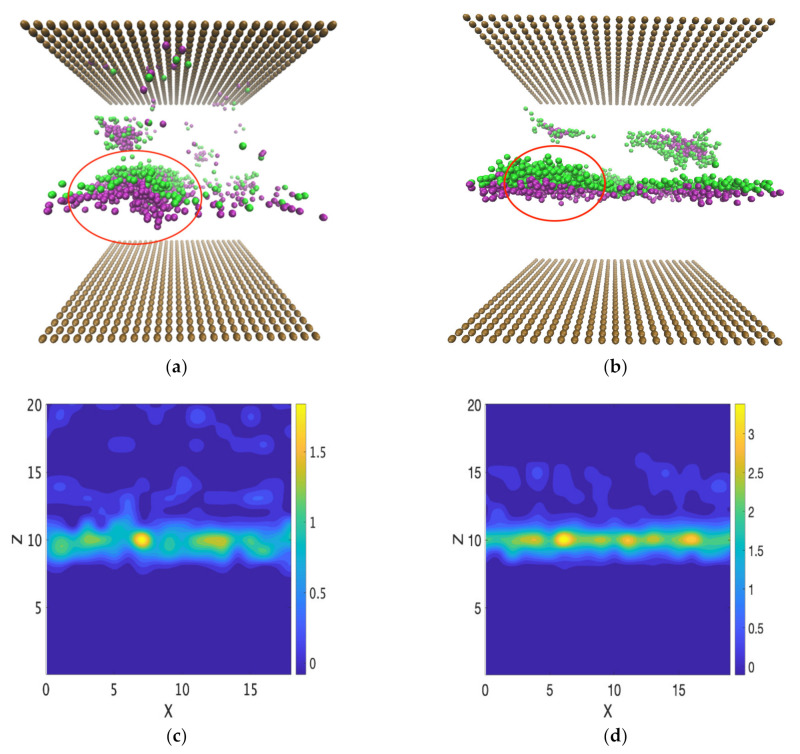
Fluid behavior of Poiseuille flow for (**a**) oil–SDS–water ($C_{SDS}=$ 50.3% CMC; $g_{x}$ = 0.09) and (**b**) oil–C12E8–water ($C_{C12E8}=$ 55.1% CMC; $g_{x}$ = 0.17). The color code for the DPD beads follows the colors in Figure 6. Water and oil beads have been hidden. Note the high local concentration of the surfactants in the circled areas. The contour plots of the density of SDS surfactant and C12E8 surfactant are presented in (**c**,**d**) to show the local increase in surfactant concentration along the interface.

To quantify the effect of surfactant concentration on the critical shear rate for the formation of micelles under Poiseuille and Couette flows, we modified the concentration of the SDS and C12E8 at the interface. For Poiseuille flow, when the concentration of surfactants located at the interface was reduced, we still observed the formation of micelles. The reason is that Poiseuille flow between two parallel plates can be unstable as the fluid is driven by a pressure gradient and there is a possibility of generating waves at the interface between two different viscous and immiscible fluids [34]. At the CMC, micelles can be formed and the micelles partition into the water phase. However, why do we observe surfactant desorption even when the surfactant concentration is below the CMC at the interface? The answer is that the concentration increases locally. As can be seen from Figure 10, the waves at the interface between the two fluids generate a change in the local concentration of the surfactant. The surfactant molecules tend to concentrate downstream from the crest of the wave, the concentration can locally become comparable to the CMC, and then micelles can partition in the water phase (see Figure 10a,b for SDS and C12E8 behavior, respectively). When the interface is unstable, surfactants tend to assemble locally (see Figure 10c,d). Once the concentration becomes large enough, desorption and micelle formation will occur, even when the surfactant concentration is lower than that at CMC.

For Couette flow, we did not find any micelle formation when the concentration of the surfactant was lower than CMC. Plane Couette flow is unconditionally stable based on linear stability analysis for single phase flows, and has also been found to be stable for multiphase flows. In other words, since the Couette flow is more stable, the waves do not form and the surfactants do not aggregate in one location to create micelles [34].

DPD simulation has been criticized for artifacts, especially in dynamic behavior as the Schmidt number in standard DPD is close to 1, which is ideal for gases. An alternative to standard DPD has been suggested as LA-DPD [35,36], but using the standard DPD does not appear to be the reason for micelle formation below the CMC observed herein. For no flow or for Couette flow, the surfactants did not aggregate in one location of the interface to create micelles. However, for Poiseuille flow, the surfactants tended to assemble, and once the local concentration became large enough, desorption in the form of micelles was observed. Hence, micelles can appear if the concentration is comparable to the CMC locally.

#### 3.3.3. The Effect of Computational Box Size

**Figure 11:**
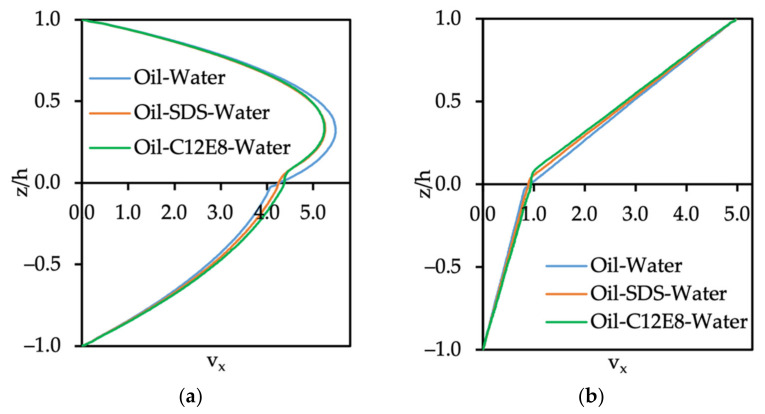
Velocity profile of the SDS or C12E8 surfactant at the interface of oil–water flow with a computational box size of $20\times 20\times 51\;r_{c}^{3}$ under (**a**) Poiseuille flow and (**b**) Couette flow configurations.

The effects of the computational box size on the fluid velocity were investigated by increasing the height of the nanoslit. The reason for probing the channel height effects was the fact that the C12E8 molecule is rather long (6 DPD units) relative to the channel height (20.8 DPD units) and the number of surfactant beads in the computation affects the viscosity of the oil or the water. The computational box height was increased from $L_{z}=21$ to $L_{z}=51$, and oil–water flow with the presence of surfactants was simulated. The same external body force as in the case of the small box ($L_{z}=21$), $g_{x}=0.01$, was imposed on each fluid particle in the x-direction to drive the Poiseuille flow. The top wall was set to move with velocity v=5.0 to create the Couette flow. In Figure 11, it can be seen that the shape of the velocity profile looks like the one in the smaller box.

For Poiseuille flow, there was about a six-fold increase in the value of the velocity at the interface (at z=0) in the case of SDS added on the oil–water interface when $L_{z}$ increased from 21 to 51. The maximum velocity of the Poiseuille flow depends on the height of the channel, as can be seen in Equations (10) and (11). For Couette flow, the larger size of the box in the z-direction did not have a significant effect on the magnitude of velocity, as expected from Equations (12) and (13). In addition, the value of velocity in the oil phase in the system of oil–C12E8–water was higher than the velocity of the oil–SDS–water system, hence, this result is the same as the one we discussed previously (small box: $20\times 20\times 21r_{c}^{3}$).

**Figure 12:**
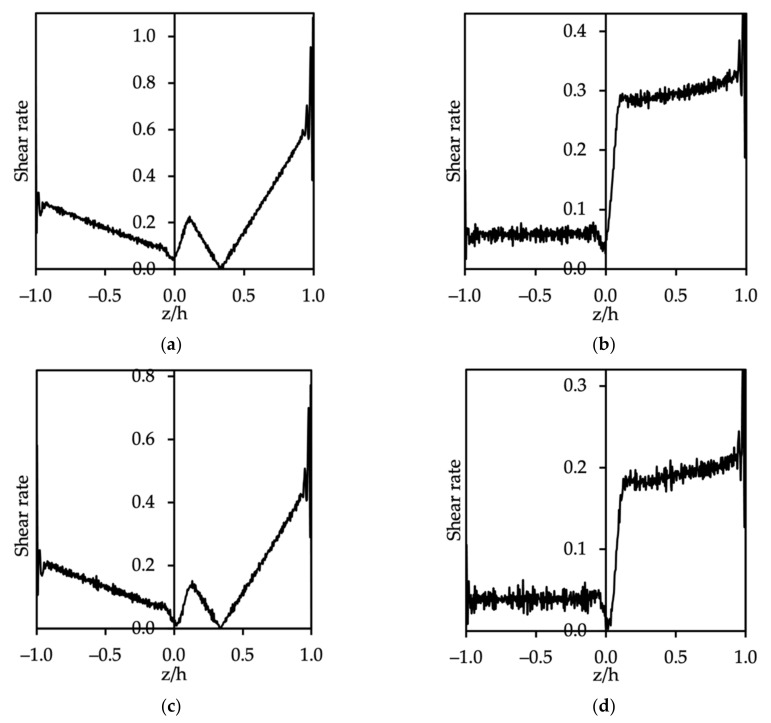
Shear rate and fluid behavior of Poiseuille flow for (**a**) oil–SDS–water and (**c**) oil–C12E8–water, and Couette flow for (**b**) oil–SDS–water and (**d**) oil–C12E8–water (box size: $20\times 20\times 51\;r_{c}^{3}$). Oil flows through the left side of the channel and water through the right side of the channel. The inset is a density plot with the y-axis as the density of the oil–surfactant–water system.

We also investigated the formation of micelles in the big box ($20\times 20\times 51r_{c}^{3}$). The SDS surfactant desorbed from the interface when the shear rate increased up to 0.226 (as shown in Figure 12a) under the Poiseuille flow condition, while Figure 12b shows that the shear effect in Couette flow led to elongated shapes for the micelles, which did not appear in the case of atop wall velocity of less than 8.8 (shear rate for oil phase was 0.058). For C12E8, as can be seen from Figure 12c, the critical shear rate was 0.151, equivalent to the external body force $g_{x}=0.008$. In addition, the top wall moved with a velocity of 5.6 (Couette flow), corresponding to a shear rate of approximately 0.039 for the oil phase, while it increased abruptly at the oil–water interface and went up slightly within the water phase (see Figure 12d). As a result, the formation of micelles in the water phase was also observed when surfactants were present in the larger simulation box.

## 4. Summary and Conclusions

In summary, we investigated the multi-fluid dynamic behavior of immiscible fluids subject to Poiseuille or Couette flow, and the interaction of surfactants with the oil–water interface under shear using the DPD simulation method. The no-slip boundary condition was satisfied by using frozen wall particles and the bounce-back boundary condition. In this way, the fluid–wall interface did not allow any water or oil beads to penetrate the wall. Moreover, the dissipative parameter for each fluid should be different to model the difference in the viscosity of the fluids before conducting simulations of the multiphase system under flow.

Selecting the appropriate dissipation parameters for the DPD model for each fluid is important. When successful, an accurate model for the viscosity of each of the two different fluids led to results that were in excellent agreement with the analytical solution of Bird et al. [22] for the case of ~50% oil and water. In addition, the results for other cases with different ratios of oil to water agreed quite well with the theoretical predictions from Equations (10)–(13).

Under shear flow, the presence of surfactants located at the oil–water interface affected the velocity profile. The presence of the surfactants modified the effective viscosity of the oil phase and water phase and led to change in the fluid velocity for the same pressure drop. Furthermore, it was demonstrated that the surfactants could desorb from the interface when the shear rate increased above a critical value. The shear led to an unstable oil–water interface in Poiseuille flow, leading to local increases in the surfactant concentration followed by surfactant desorption into the aqueous phase. Plane Couette flow was stable for immiscible fluids, so localized increases in the surfactant concentration did not occur in that case. The phenomenon of micelle formation below CMC only occurred in the case of Poiseuille flow because of the appearance of waves and the resulting increase in local surfactant concentration. Finally, it must be noted that the effects of viscosity and shear rate in the case for surfactants were confirmed with calculations in a simulation box that was twice as high.

## Figures and Tables

**Table 1:** Scaling factors for DPD computations.

Type of Surfactant	Density	Number of Water Molecules in One Bead	Mass Scale $\left(10^{-25}\;\mathrm{kg}\right)$	Length Scale $\left(10^{-10}\;m\right)$	Temperature Scale (K)	Time Scale $\left(10^{-12}\;s\right)$
SDS	5	5	1.5	9.09	298	5.48
C12E8	5	6	1.8	9.66	298	6.38
None	5	5	1.5	9.09	298	5.48

**Table 2:** Interaction parameters ($a_{ij}$) used in the DPD computations.

	W	O	Wall
**W**	15	90	15
**O**		15	20
**Wall**			15

**Table 3:** Set of repulsion parameters used in the water–oil–SDS simulations. H and T represent the head and tail beads of the SDS surfactant, while W and O denote the water and oil, respectively.

	H	T	W	O	Wall
**H**	20	42	10	50	35
**T**		15	25	12	15.5
**W**			15	90	15
**O**				15	20
**Wall**					15

**Table 4:** Details of parameters used in the water–oil–C12E8 simulations. H and T represent the head and tail of the C12E8 surfactant, while W and O denote the water and oil, respectively.

	H	T	W	O	Wall
**H**	15	25	14	25	35
**T**		15	54	14.5	15.5
**W**			15	100	15
**O**				15	20
**Wall**					15

**Table 5:** Errors for two-phase flow velocity profile.

**Percentage of oil**	0.0%	28.8%	45.4%	65.6%	100.0%
$\mathrm{Normalized}\;\mathrm{error}\;{\bar{e}}_{L_{2}}$ **(Poiseullie flow)**	0.024	0.023	0.032	0.029	0.022
$\mathrm{Normalized}\;\mathrm{error}\;{\bar{e}}_{L_{2}}$ **(Couette flow)**	0.007	0.009	0.017	0.021	0.007

## Data Availability

The data presented in this study are available on request from the corresponding author.

## References

[1] Soares E.J., Thompson R.L. (2009). Flow regimes for the immiscible liquid–liquid displacement in capillary tubes with complete wetting of the displaced liquid. J. Fluid Mech..

[2] Zhao H., Ning Z., Kang Q., Chen L., Zhao T. (2017). Relative permeability of two immiscible fluids flowing through porous media determined by lattice Boltzmann method. Int. Commun. Heat Mass Transf..

[3] Li J., Sheeran P.S., Kleinstreuer C. (2011). Analysis of multi-layer immiscible fluid flow in a microchannel. J. Fluids Eng..

[4] Gaddam A., Garg M., Agrawal A., Joshi S.S. (2015). Modeling of liquid–gas meniscus for textured surfaces: Effects of curvature and local slip length. J. Micromech. Microeng..

[5] Tang X., Xiao S., Lei Q., Yuan L., Peng B., He L., Luo J., Pei Y. (2018). Molecular dynamics simulation of surfactant flooding driven oil-detachment in nano-silica channels. J. Phys. Chem. B.

[6] Fan J.C., Wang F.C., Chen J., Zhu Y.B., Lu D.T., Liu H., Wu H.A. (2018). Molecular mechanism of viscoelastic polymer enhanced oil recovery in nanopores. R. Soc. Open Sci..

[7] Karaborni S., Van Os N., Esselink K., Hilbers P.A.J. (1993). Molecular dynamics simulations of oil solubilization in surfactant solutions. Langmuir.

[8] Smit B., Schlijper A., Rupert L., Van Os N. (1990). Effects of chain length of surfactants on the interfacial tension: Molecular dynamics simulations and experiments. J. Phys. Chem..

[9] Karniadakis G., Beskok A., Aluru N. (2006). Microflows and Nanoflows: Fundamentals and Simulation.

[10] Rezaei H., Modarress H. (2015). Dissipative particle dynamics (DPD) study of hydrocarbon–water interfacial tension (IFT). Chem. Phys. Lett..

[11] Pivkin I.V., Karniadakis G.E. (2005). A new method to impose no-slip boundary conditions in dissipative particle dynamics. J. Comput. Phys..

[12] Xu Z., Meakin P. (2009). A phase-field approach to no-slip boundary conditions in dissipative particle dynamics and other particle models for fluid flow in geometrically complex confined systems. J. Chem. Phys..

[13] Soares J.S., Gao C., Alemu Y., Slepian M., Bluestein D. (2013). Simulation of platelets suspension flowing through a stenosis model using a dissipative particle dynamics approach. Ann. Biomed. Eng..

[14] Backer J., Lowe C., Hoefsloot H., Iedema P. (2005). Poiseuille flow to measure the viscosity of particle model fluids. J. Chem. Phys..

[15] Belhaj A.F., Elraies K.A., Mahmood S.M., Zulkifli N.N., Akbari S., Hussien O.S. (2020). The effect of surfactant concentration, salinity, temperature, and pH on surfactant adsorption for chemical enhanced oil recovery: A review. J. Pet. Explor. Prod. Technol..

[16] Vu T.V., Papavassiliou D.V. (2018). Oil-water interfaces with surfactants: A systematic approach to determine coarse-grained model parameters. J. Chem. Phys..

[17] Vu T.V., Razavi S., Papavassiliou D.V. (2022). Effect of Janus particles and non-ionic surfactants on the collapse of the oil-water interface under compression. J. Colloid Interface Sci..

[18] Green D.W., Willhite G.P., Henry L. (1998). Enhanced Oil Recovery.

[19] Shah D.O. (2012). Improved Oil Recovery by Surfactant and Polymer Flooding.

[20] Hoogerbrugge P., Koelman J. (1992). Simulating microscopic hydrodynamic phenomena with dissipative particle dynamics. EPL (Europhys. Lett.).

[21] Groot R.D., Warren P.B. (1997). Dissipative particle dynamics: Bridging the gap between atomistic and mesoscopic simulation. J. Chem. Phys..

[22] Espanol P., Warren P. (1995). Statistical mechanics of dissipative particle dynamics. EPL (Europhys. Lett.).

[23] Bird R.B., Stewart W.E., Lightfoot E.N. (2006). Transport Phenomena.

[24] Plimpton S. (1995). Computational limits of classical molecular dynamics simulations. Comput. Mater. Sci..

[25] Groot R.D., Rabone K. (2001). Mesoscopic simulation of cell membrane damage, morphology change and rupture by nonionic surfactants. Biophys. J..

[26] Vu T.V., Papavassiliou D.V. (2018). Modification of Oil–Water Interfaces by Surfactant-Stabilized Carbon Nanotubes. J. Phys. Chem. C.

[27] Vo M., Papavassiliou D.V. (2016). Interaction parameters between carbon nanotubes and water in Dissipative Particle Dynamics. Mol. Simul..

[28] Humphrey W., Dalke A., Schulten K. (1996). VMD: Visual molecular dynamics. J. Mol. Graph..

[29] Doolittle A.K. (1951). Studies in Newtonian flow. II. The dependence of the viscosity of liquids on free-space. J. Appl. Phys..

[30] Pan D., Phan-Thien N., Khoo B.C. (2014). Dissipative particle dynamics simulation of droplet suspension in shear flow at low Capillary number. J. Non-Newton Fluid.

[31] Mondello M., Grest G.S. (1997). Viscosity calculations of n-alkanes by equilibrium molecular dynamics. J. Chem. Phys..

[32] Doi M., Edwards S.F. (1986). The Theory of Polymer Dynamics.

[33] Nissan A., Grunberg L. (1949). Mixture law for viscosity. Nature.

[34] Charru F., Fabre J. (1994). Long waves at the interface between two viscous fluids. Phys. Fluids.

[35] Prhashanna A., Khan S.A., Chen S.B. (2016). Micelle morphology and chain conformation of triblock copolymers under shear: LA-DPD study. Colloids Surf. A Physicochem. Eng. Asp..

[36] Yaghoubi S., Shirani E., Pishevar A., Afshar Y. (2015). New modified weight function for the dissipative force in the DPD method to increase the Schmidt number. EPL (Europhys. Lett.).

